# Genome-Centric Analysis of a Thermophilic and Cellulolytic Bacterial Consortium Derived from Composting

**DOI:** 10.3389/fmicb.2017.00644

**Published:** 2017-04-19

**Authors:** Leandro N. Lemos, Roberta V. Pereira, Ronaldo B. Quaggio, Layla F. Martins, Livia M. S. Moura, Amanda R. da Silva, Luciana P. Antunes, Aline M. da Silva, João C. Setubal

**Affiliations:** ^1^Departamento de Bioquímica, Instituto de Química, Universidade de São PauloSão Paulo, Brazil; ^2^Programa de Pós-Graduação Interunidades em Bioinformática, Universidade de São PauloSão Paulo, Brazil; ^3^Biocomplexity Institute, Virginia TechBlacksburg, VA, USA

**Keywords:** bacterial genome reconstruction, consortium, thermophilic, cellulolytic, glycoside hydrolases, composting, metagenome

## Abstract

Microbial consortia selected from complex lignocellulolytic microbial communities are promising alternatives to deconstruct plant waste, since synergistic action of different enzymes is required for full degradation of plant biomass in biorefining applications. Culture enrichment also facilitates the study of interactions among consortium members, and can be a good source of novel microbial species. Here, we used a sample from a plant waste composting operation in the São Paulo Zoo (Brazil) as inoculum to obtain a thermophilic aerobic consortium enriched through multiple passages at 60°C in carboxymethylcellulose as sole carbon source. The microbial community composition of this consortium was investigated by shotgun metagenomics and genome-centric analysis. Six near-complete (over 90%) genomes were reconstructed. Similarity and phylogenetic analyses show that four of these six genomes are novel, with the following hypothesized identifications: a new *Thermobacillus* species; the first *Bacillus thermozeamaize* genome (for which currently only 16S sequences are available) or else the first representative of a new family in the Bacillales order; the first representative of a new genus in the Paenibacillaceae family; and the first representative of a new deep-branching family in the Clostridia class. The reconstructed genomes from known species were identified as *Geobacillus thermoglucosidasius* and *Caldibacillus debilis*. The metabolic potential of these recovered genomes based on COG and CAZy analyses show that these genomes encode several glycoside hydrolases (GHs) as well as other genes related to lignocellulose breakdown. The new *Thermobacillus* species stands out for being the richest in diversity and abundance of GHs, possessing the greatest potential for biomass degradation among the six recovered genomes. We also investigated the presence and activity of the organisms corresponding to these genomes in the composting operation from which the consortium was built, using compost metagenome and metatranscriptome datasets generated in a previous study. We obtained strong evidence that five of the six recovered genomes are indeed present and active in that composting process. We have thus discovered three (perhaps four) new thermophillic bacterial species that add to the increasing repertoire of known lignocellulose degraders, whose biotechnological potential can now be investigated in further studies.

## Introduction

Plant biomass can be decomposed by complex lignocellulolytic microbial communities present in natural environments, such as forest soil (Eichorst and Kuske, 2012) and cow rumen (Hess et al., 2011), or in engineered ecosystems, such as composting (Neher et al., 2013) or biogas fermenters (Güllert et al., 2016). For biomass degradation in these environments lignocellulolytic fungal and bacterial species employ hydrolytic and oxidative enzymes, which act synergistically to depolymerize cellulose, hemicellulose, and lignin (Allgaier et al., 2010; Koeck et al., 2014; Hemsworth et al., 2015; López-Mondéjar et al., 2016). These studies have thus shown that taxonomically diverse members within a lignocellulolytic microbial community work in cooperation to fully deconstruct plant biomass.

Microbial consortia selected from complex lignocellulolytic microbial communities are promising alternatives to deconstruct plant waste rather than microbial monocultures (Zuroff and Curtis, 2012; Peng et al., 2016). Indeed, culture enrichment provides a good strategy for studying the interactions among their microbial members as well as their lignocellulolytic enzyme systems (Wongwilaiwalin et al., 2010; D'Haeseleer et al., 2013; Kinet et al., 2015; de Lima Brossi et al., 2016; Zhu et al., 2016). Moreover, natural microbial consortia can be a method of culture enrichment for unculturable microbial species (Vartoukian et al., 2010; D'Haeseleer et al., 2013).

Several studies have succeeded in establishing lignocellulolytic consortia from diverse sources such as soil (Feng et al., 2011; Gao et al., 2014; Jiménez et al., 2014; de Lima Brossi et al., 2016; Cortes-Tolalpa et al., 2016), biogas-producing digester (Yan et al., 2012) and composting (Haruta et al., 2002; Wongwilaiwalin et al., 2010; Gladden et al., 2011; Eichorst et al., 2013; Kinet et al., 2015; Wang et al., 2016; Zhu et al., 2016). Even though all these consortia exhibit lignocellulose degradation capabilities, their microbial community structure, and composition are distinct. The composition of the final microbial consortia appears to be strongly driven by the substrate used as carbon source (Eichorst et al., 2014; Simmons et al., 2014b; de Lima Brossi et al., 2016), the nutrient availability (complex or minimal medium; Mello et al., 2016) and by the inoculum source (Cortes-Tolalpa et al., 2016).

Shotgun metagenomics is a powerful approach to investigate the lignocellulose-degrading potential of microbial communities and has substantially expanded the repertoire of genes and genomes related to plant biomass decomposition (D'Haeseleer et al., 2013; Jimenez et al., 2015; Mhuantong et al., 2015; Antunes et al., 2016; Wang et al., 2016). Furthermore, advances in multi-omics approaches are improving our capacity to explore and mine new biomass-degrading genes (Mhuantong et al., 2015). This approach has also allowed bacterial genome reconstruction of novel microbial species from natural and engineered ecosystems, favoring detailed genome-centric exploration of microbial communities (Albertsen et al., 2013; D'Haeseleer et al., 2013; Rosewarne et al., 2013; Delmont et al., 2015; Nelson et al., 2015; Antunes et al., 2016; Gupta et al., 2016; Sangwan et al., 2016; Stolze et al., 2016; Vanwonterghem et al., 2016). A genome-centric approach is one that emphasizes the analysis of complete or near-complete genomes, as opposed to catalogs of genes based on sequencing read analysis. The advantages of using a genome-centric analysis are that it provides more extensive data on which to base phylogenetic identifications (Hug et al., 2016) and the establishment and quantification of entire gene repertories in single organisms (Prosser, 2015), thereby allowing the possibility of a fuller understanding of individual microbial physiologies as well as their relationship to other organisms sharing the same ecological niche (Albertsen et al., 2016; Li et al., 2016). Based on a literature survey (Albertsen et al., 2013; D'Haeseleer et al., 2013; Delmont et al., 2015; Nelson et al., 2015; Antunes et al., 2016; Stolze et al., 2016; Vanwonterghem et al., 2016) we estimate that the number of reconstructed genomes with at least 90% completeness obtained from biomass degrading environments currently available is about 60. Given the compositional complexity of these environments, it is probably the case that thousands more additional novel species await discovery through genome reconstruction techniques applied to shotgun metagenome sequencing data.

Here, we have used shotgun metagenomics to investigate the microbial community composition of a thermophilic aerobic consortium enriched through multiple passages at 60°C in carboxymethylcellulose (CMC) as exogenous carbon source, using São Paulo Zoo Park compost as the inoculum. In previous studies (Martins et al., 2013; Antunes et al., 2016) we showed that this thermophilic composting operation harbors an impressive variety of bacterial species and metabolic functions related to biomass degradation; that result was the primary motivation for this work. The metagenomic sequences derived from the bacterial consortium under study allowed us to recover six near-complete genomes. We provide putative identifications for these six genomes, showing that at least three of them appear to be novel species. We also provide a detailed analysis of the biomass-degrading gene content in each genome.

## Materials and methods

### Preparation of a composting-derived microbial consortium

The inoculum for consortium preparation derived from a composting facility of São Paulo Zoo Park, São Paulo, Brazil. The sample was collected on October/2013, after 30 days from the beginning of the composting process. Composting pile temperature was in the range of 70–72°C. The São Paulo Zoo composting facility is designed to compost 4 tons/day of all organic waste produced in the park comprising mainly shredded tree branches, leaves and grass from the maintenance of park green areas, plus manure, beddings, and food residues from about 400 species of zoo animals (mammals, avian, and reptiles). The composting process last ~100 days and is performed according a well-defined procedure (Martins et al., 2013; Antunes et al., 2016).

The compost sample was transported to the laboratory in a thermic box and immediately processed. A suspension was prepared by adding 10 g of the compost sample to 50 mL Falcon tube containing 25 mL of NaCl 0.9%. The tube was statically incubated at 60°C for 48 h. The mixture was filtered through layers of sterile cotton gauze and 150 μL of the filtrate were inoculated in 10 mL of M9 minimal medium supplemented with 1% CMC. The tube was statically incubated at 60°C for ~7–10 days, until loss of medium viscosity could be visually inspected as an indication of CMC consumption. The microbial cells were pelleted by centrifugation (8,000 × g) and ressuspended in 1 mL of fresh M9 minimal medium and 100 μL aliquot of this cell suspension were transferred to 10 mL of fresh medium supplemented with 1% CMC. The procedure was repeated for seven times, resulting in eight sequential passages to generate the final thermophilic consortium. M9 minimal medium without CMC was used as control in the sequential passages, and no detectable microbial growth was observed. Aliquots (1 mL) of the final consortium that was named ZCTH02 were stored at −80°C in 20% glycerol or further processed for total DNA extraction and shotgun metagenomic sequencing.

### Shotgun metagenomic sequencing

The DNA extraction from the ZCTH02 culture (1 mL-aliquot) was performed with MoBio DNA Power Soil kit (MoBio Laboratories, Carlsbad, USA) according to the manufacturer's instructions. Purity and concentration of DNA were evaluated on a ND-1000 spectrophotometer (Nano Drop Technologies, Wilmington, USA). Additional quantification was performed with Quant-iT Picogreen dsDNA assay kit (Life Technologies, Grand Island, USA). DNA integrity was verified using 2100 Bioanalyzer DNA 7500 chip (Agilent Technologies, Santa Clara, USA). Shotgun metagenomic library was generated using the Illumina Nextera DNA library preparation kit (Illumina, Inc., San Diego, USA) as recommended by the manufacturer using 35 ng of total DNA. Size and quality of DNA fragment library were assessed using 2100 Bioanalyzer Agilent High Sensitivity DNA chip. Library quantification with KAPA Library Quantification Kit (Kapa Biosystems, Wilmington, USA), normalization, dilution and pooling were performed following standard protocols for sequencing in the Illumina MiSeq platform (Illumina, Inc., CA). Sequencing run was performed the MiSeq Reagent kit v2 (500-cycle format, paired-end (PE) reads). Illumina PE read1 and read2 presented, respectively, >75 and >70% of bases with quality score above phred 30 (Q30).

### Processing, quality control, and assembly of the metagenomic sequences

Raw PE sequencing reads were quality-filtered to remove reads shorter than 150 bp and reads with average quality score lower than phred 30 using SICKLE and default parameters (Joshi and Fass, 2011). Although these parameters are restrictive, they warrant better accuracy in the metagenomic assembly process (Sharon et al., 2015). Assembly of merged quality-filtered paired-end reads was performed with SPAdes using Illumina paired end reads and multi-cell data set parameters (Nurk et al., 2013). Contigs smaller than 1,600 bp were removed from downstream analyses. The coverage of each contig was calculated by mapping all high quality reads back to the final assembly by using Bowtie2 (Langmead and Salzberg, 2012).

### Genome reconstruction based on binning methods

The reconstruction of individual genomes was performed with MaxBin software (Wu et al., 2014). This approach combines tetranucleotide frequencies and contig coverage to cluster metagenomic contigs into individual bins, which were validated by the identification of marker genes for each bin. The quality control of the reconstructed genomes was evaluated with CheckM (Parks et al., 2015).

### Taxonomic assignment and phylogenetic analysis

For taxonomic assignment and phylogenetic analysis of each reconstructed genome, a strategy based on two steps was employed. The first step was based on the comparative analysis of 16S rRNA and a single gene marker (Albertsen et al., 2013) between nr database using BLAST (Altschul et al., 1997). After the identification of similar microorganisms with each individual genome, the second step was to select phylogenetically close microbial genomes and apply the phylogenetic analysis. Phylogenetic reconstruction was conducted using PhyloPhlAn software (Segata et al., 2013) and trees were generated using iTol (Letunic and Bork, 2016). Digital DNA-DNA hybridization (dDDH) was used to compare genome-to-genome similarity and each reconstructed genome was compared to their closest genome using GGDC tool (Auch et al., 2010).

### Functional annotation

Genomes were annotated using an upgraded version of the NCBI Prokaryotic Genome Automatic Annotation Pipeline (Angiuoli et al., 2008). Protein sequences were compared against Clusters of Orthologous Groups (COGs) database (Galperin et al., 2015) using rpsblast+ (Altschul et al., 1997), with a cut-off *e* < 10^−2^. COG categories were assigned for the best hits with cdd2cog script^1^.

The amino acid sequences of the predicted coding sequences CDSs were screened using the dbCAN HMM-based database (Yin et al., 2012) for carbohydrate-active enzymes (CAZymes; Lombard et al., 2014) and protein domains implicated in lignocellulose degradation with HMMER package (Eddy, 2009). If alignment length >80 amino acids, the cut-off *e*-value used was 10^−5^, otherwise 10^−3^ was used. Data visualization was performed using Circos software (Krzywinski et al., 2009).

### Mapping of reconstructed genomes against metagenomic and metatranscriptomic datasets

Sequencing reads from previous metagenomics and metatranscriptomics studies of the thermophilic composting cell ZC4 (Antunes et al., 2016) were used for mapping. These datasets consist of nine time-series samples from composting cell ZC4 (days 1, 3, 7, 15, 30, 64, 67, 78, and 99). These sequencing reads were mapped against the reconstructed genomes to investigate their presence and activity in ZC4. For the purposes of determining the presence of reconstructed genomes in ZC4 data, we computed genome coverage under the following definition. A given base pair position in a contig that is part of a reconstructed genome was considered covered if at least one ZC4 sequencing read was mapped to that position. This computation was done using program genomecov in package BEDTools (Quinlan and Hall, 2010). For the purposes of determining relative abundance of reconstructed genomes in the metagenomic datasets we mapped the sequencing reads against the reconstructed contig sequences. For the purposes of determining relative abundance of reconstructed genomes in the metatranscriptomic datasets we mapped the sequencing reads against coding sequence regions annotated in each reconstructed genome. Abundance was measured in terms of number of reads mapped. Mapping was performed with BBMap^2^. Only reads with length greater or equal to 100 bp were taken into account. A mapping was considered valid if the alignment had at least 95% identity.

### Accession numbers

The recovered genomes were deposited in GenBank under the following accession numbers: LZRU00000000 (BZ1), LZRT00000000 (BZ2), LZRV00000000 (BZ3), LZRS00000000 (BZ4), SAMN05223358 (BZ5), and LZRQ00000000 (BZ6). Unassembled paired-end sequence reads of ZCTH02 metagenome are available at MG-RAST under the under the accession mgm4570098.3 (read1) and mgm4570099.3 (read2).

## Results

### Reconstruction of six genomes from a compost-derived bacterial consortium

A total of 3,046,968 sequence paired-end reads was obtained from a thermophilic and cellulolytic consortium (ZCTH02; Table 1). Taxonomic analysis of all protein-coding sequences of the ZCTH02 metagenome at the MG-RAST webserver (Keegan et al., 2016) show that it is dominated by Bacteria (99% of total sequences), most of which (86%) are classified as Firmicutes. At the order level, the consortium metagenome protein-coding sequences were identified as belonging primarily to Bacillales (72%) and Clostridiales (10%).

**Table 1:** **Assembly metrics of shotgun metagenomics of the ZCTH02 consortium**.

**Parameter**	**ZCTH02 consortium**
Sample description	Thermophilic and cellulolytic composting-derived bacterial consortium
Location of São Paulo Zoo composting (source of inoculum)	23°38′56.9″S 46°37′18.7″W
Metagenome ID	ZCTH02
Number of paired-end reads	3,046,968
Number of assembled contigs	13,240
N50 (bp)	17,996
Longest contig (bp)	509,962
Total assembled length (bp)	27,862,858
Number of assembled contigs ≥1,600 bp^a^	1,468
Number of paired-end reads in contigs ≥1,600 bp^a^	1,263,585
Total assembled contigs length ≥1,600 bp (bp)^a^	20,608,826

a *Only this dataset was used on binning analysis*.

The metagenome shotgun paired-end reads were assembled into 1,468 contigs ≥1,600 bp, comprising 1,263,585 paired-end reads totalizing 0.206 Gbp (Table 1). Using 97% of these contigs we were able to reconstruct six nearly-complete bacterial genomes, which were named BZ1, BZ2, BZ3, BZ4, BZ5, and BZ6 (Table 2). We remark that the length-filtering parameter used during the assembly steps was quite stringent, in order to minimize the presence of chimeric contigs. Quality control (Parks et al., 2015) on the resulting genomes yielded the following values: at least 90% completeness and <5% contamination in all cases.

**Table 2:** **Genomic features of bacterial genomes reconstructed from the ZCTH02 consortium shotgun metagenome**.

**Genome name**	**BZ1**	**BZ2**	**BZ3**	**BZ4**	**BZ5**	**BZ6**
Hypothesis	New *Thermobacillus* species	First *Bacillus thermozeamaize* genome	New Paenibacillaceae genus (similar to *Paenibacillus* and *Cohnella*) genome	*Geobacillus thermoglucosidasius* genome	*Caldibacillus debilis* genome	New deep-branching family (Clostridia class) genome
Estimated Genome Size (Mb)	3.37	3.44	3.12	4.38	2.86	2.77
Number of contigs	46	143	187	244	244	304
Mapped reads (%)	32.85	22.18	15.75	13.86	7.34	7.40
GGDC (Difference in % G+C)^a^	3.77 (distinct species)	2.05 (interpretation: distinct species with respect to *Caldibacillus debillis*)	3.77 (distinct species)	0.54 (either distinct or same species)	0.52 (either distinct or same species)	15.40 (distinct species)
Best hit (16S rRNA)^b^ (NCBI Accession number)	Uncultured bacteria (FN687454)	*Bacillus thermozeamaize* (AY288912.1)	Uncultured bacterium (JQ775380.1)	*Geobacillus thermoglucosidasius* (CP002835.1)	Uncultured *Bacillus* sp. (AB201612)	Uncultured composting bacterium (FN667161.1)
Coverage/Identity (%)	97/99	99/99	95/100	100/99	93/100	95/98
Best hit (DNA Primase) (%) - nt	*Thermobacillus composti*	*Enterococcus rotai*	*Paenibacillus* sp.	*Geobacillus thermoglucosidasius*	*Caldibacillus debilis*^d^	NA
Coverage/Identity (%)	100/89	3/83	8/79	100/99	100/100	NA^e^
Estimated Completeness (%)^c^	95.05	95.38	93.35	99.44	97.96	91.58
Estimated Contamination (%)^c^	0.00	1.41	4.92	2.20	0.58	4.83
G+C content (%)	64.34	53.68	62.51	43.41	52.15	65.53
Maximum scaffold length (bp)	509,962	208,013	120,895	122,932	49,485	62,878
N50 contig length	168,514	69,814	33,295	33,740	18,062	12,917
CDS number	3,041	3,297	2,969	4,376	3,156	2,722

a *GGDC (Genome-to-Genome Distance Calculator) comparison between thermophilic compost genomes reconstructed in this work and most similar genome*.

b *16S rRNA comparative analysis with partial bin sequences*.

c *Estimated completeness and contamination of draft genome based on single copy lineage-specific marker genes (Parks et al., 2015)*.

d *Direct comparisons between BZ5 and C. debilis (NZ_KB912918.1) genome*.

e *No hit with nt database*.

The reconstructed genomes range in size from 2.7 to 4.4 Mbp, in %GC from 43.4 to 65.5%, and in coding sequences (CDSs) from 2,969 to 4,376 (Table 2). All recovered genomes belong to the Firmicutes phylum. Measuring abundance in terms of reads actually used to assemble the 1,468 binned contigs, BZ1 was the most abundant of all six reconstructed genomes (33%), followed by BZ2 (22%).

### Taxonomic and phylogenetic assignment of reconstructed genomes

**Figure 1:**
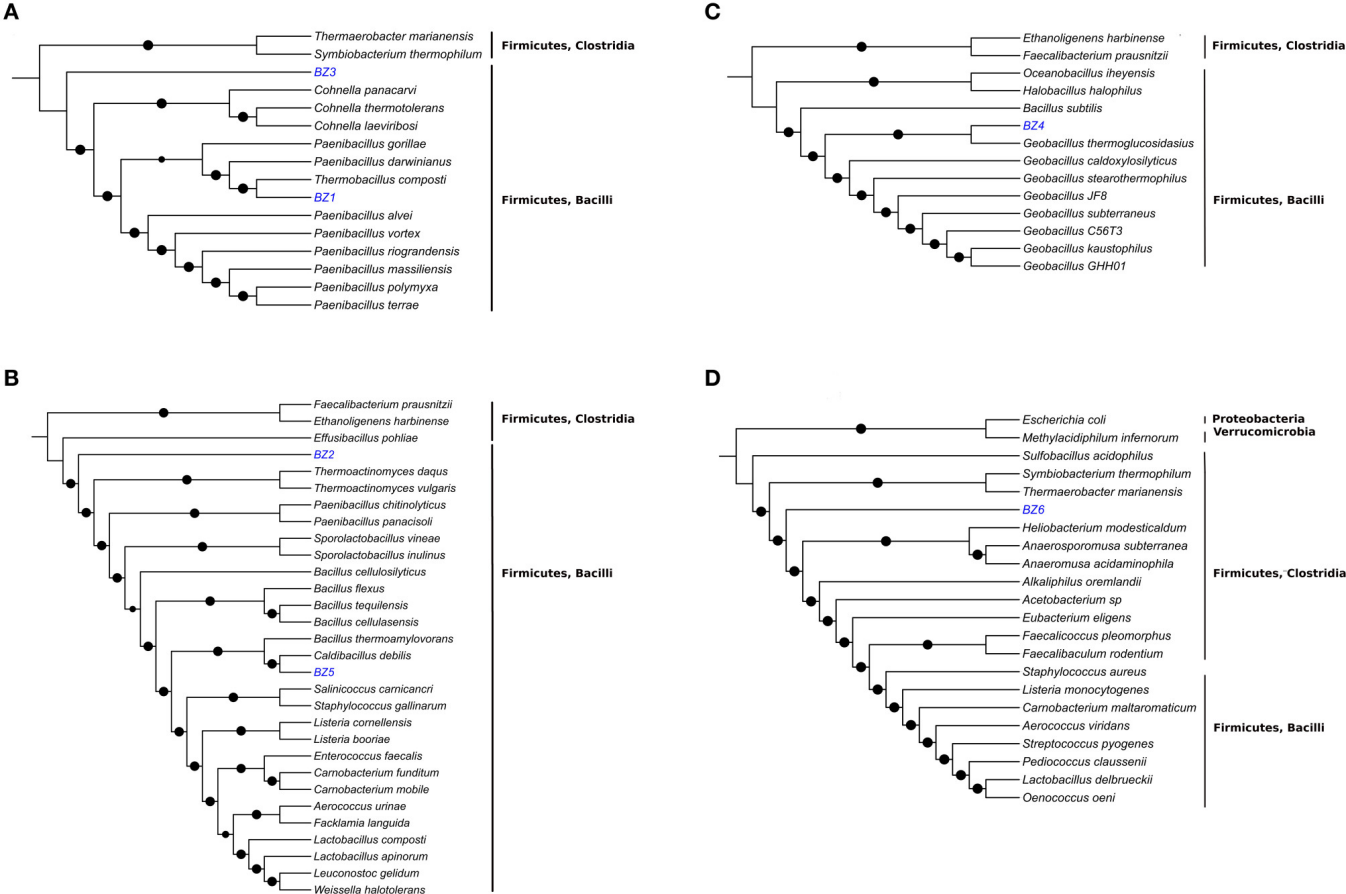
**Phylogenetic analysis of the six reconstructed bacterial genomes**. The analyses were based on ~400 conserved single-copy protein sequences, selected among microbial type strain genomes phylogenetically close to BZ1 and BZ3 **(A)**, BZ2 and BZ5 **(B)**, BZ4 **(C)**, and BZ6 **(D)**. Black dots indicate bootstrap values of ≥80%.

For each reconstructed genome, we sought to determine their taxonomic classification and phylogenetic placement using (i) BLAST similarity analysis based on 16S rRNA and DNA primase nucleotide sequences; and (ii) phylogenetic tree reconstruction based on the amino acid sequences of a small set of housekeeping genes. The BLAST results are shown in Table 2 and the phylogenetic trees are shown in Figure 1. Taxonomic and phylogenetic analyses suggest that three of the six reconstructed genomes belong to novel bacteria, with the following preliminary identifications: a new *Thermobacillus* species (BZ1); a member of a new genus of the Paenibacillaceae family (BZ3), similar to *Paenibacillus* and *Cohnella*; and a member of a new deep-branching family in the Clostridia class (BZ6). In addition, we provide a tentative identification of BZ2 as *Bacillus thermozeamaize*. If this identification is correct this reconstructed genome would be the first for this species, for which currently only 16S rRNA sequences are available. Except for BZ6, the other reconstructed genomes are members of the Bacilli class. We provide more details of these results in what follows.

For BZ1 the best 16S rRNA hit (99% identity) was to a sequence from a non-culturable microorganism found in an autothermal thermophilic aerobic digester in Australia (Hayes et al., 2011). *Thermobacillus composti* was the best hit (89% identity) for the DNA primase gene sequence. Previous studies based on 16S rRNA (Ash et al., 1993) and housekeeping genes (Zhang and Lu, 2015) have shown that the *Thermobacillus* genus is close to the *Paenibacillus* genus. Based on these results we carried out the phylogenetic reconstruction using members of the Paenabacillaceae family (Figure 1A). Comparative genome analysis based on GGDC between BZ1 and *T. composti* KWC4 reference genome suggests that they are distinct species (Difference in % G+C = 3.77). The inferred phylogenetic tree and these results indicate that BZ1 is likely a new species in the *Thermobacillus* genus.

For BZ2, the best 16S rRNA hit (99% identity) was to a sequence from *Bacillus thermozeamaize* strain L-10997 (accession AY288912.1). This strain was isolated from batch fermentations samples of thermophilic and hyperthermophilic food processing facilities (Mak, 2003). A closely related *B. thermozeamaize* isolate was also found among thermophilic cellulose-degrading cultivable bacteria from a deep subsurface gold mine (Rastogi et al., 2009). Our BLAST searches using the DNA primase gene sequence did not yield any good matches. In GenBank there are no reports of *B. thermozeamaize* genomes. Phylogenetic analysis (Figure 1B) shows that BZ2 is closer to *Effusibacillus* and *Thermoactinomyces* than to *Bacillus*. The BLAST analysis and the phylogenetic analysis point to three possible conclusions: (1) BZ2 is a *B. thermozeamaize* strain but the classification of *B. thermozeamaize* as belonging to the genus *Bacillus* is wrong; or (2) BZ2 is *not* a *B. thermozeamaize* strain, and the 16S rRNA similarity is misleading; or (3) the classification of the 16S rRNA sequence in record AY288912.1 is incorrect. If BZ2 is not a *B. thermozeamaize* strain then it would likely be the first representative of a new family in the Bacillales order.

For BZ3, the best hit for the 16S rRNA gene was with an uncultured bacterium detected in asparagus straw compost in YongJi, ShanXi, China (accession JQ775380.1); however, the query was not a complete 16S rRNA gene sequence. DNA primase analysis shows a distant relationship to genes from members of the *Paenibacillus* genus (79% identity/8% coverage). Phylogenetic analysis (Figure 1A) indicates that BZ3 is a member of the Paenibacillaceae family and might be a member of a new genus.

BZ4 and BZ5 appear to be new strains of known species. 16S rRNA and DNA primase analyses (Table 2) show that BZ4 and BZ5 are very similar to *Geobacillus thermoglucosidasius* (99% identity) and *Caldibacillus debilis* (100%), respectively. Phylogenetic analyses (Figures 1C,B) and GGDC analysis confirm this result (Table 2). The genomes for three strains of *G. thermoglucosidasius* (DSM2542, C56-YS93, and TNO-09.020) are available (Zhao et al., 2012; Brumm et al., 2015; Chen et al., 2015). *C. debilis* has a genome draft published (Berendsen et al., 2016).

**Figure 2:**
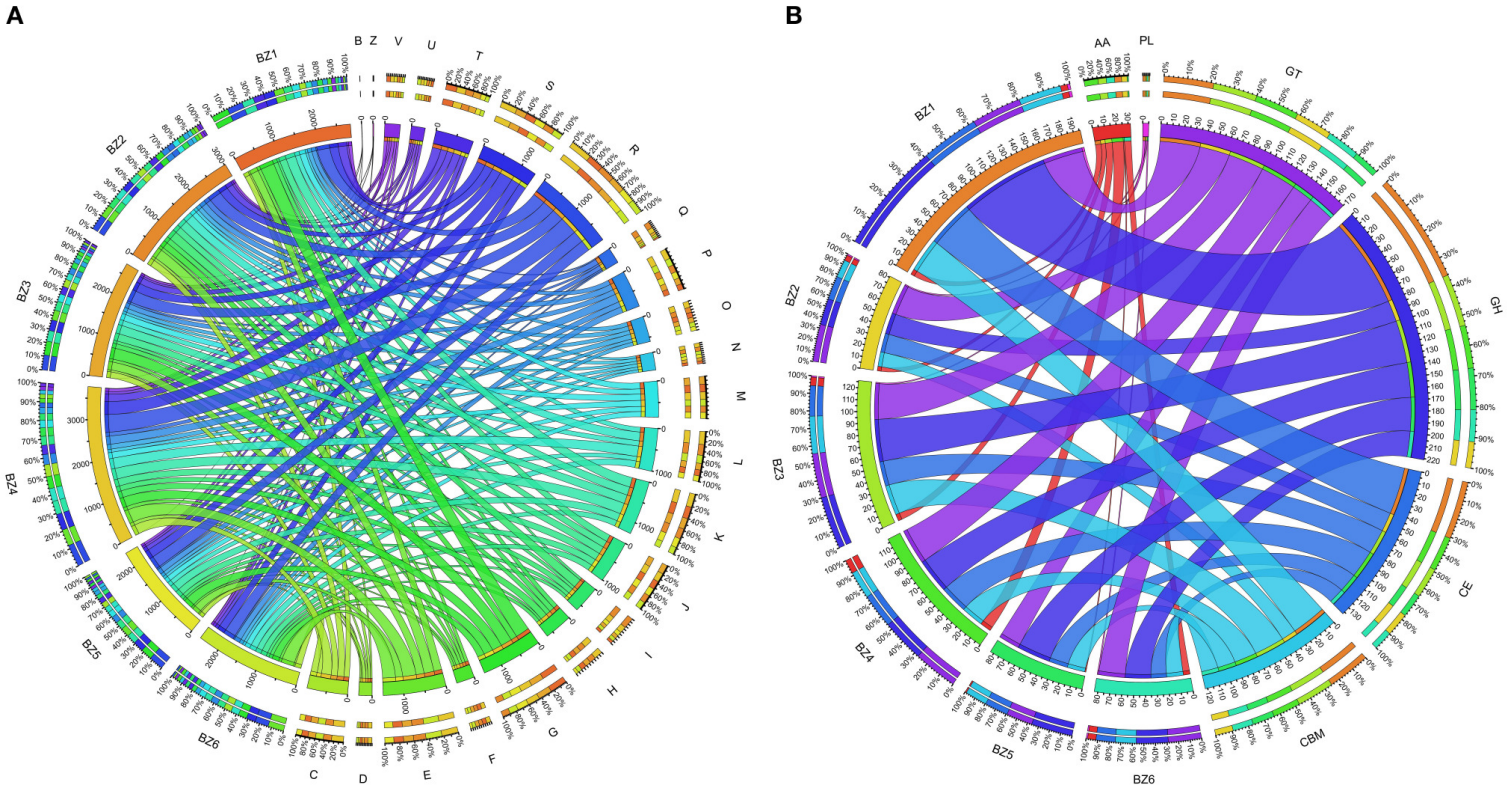
**Functional profile of the six reconstructed genomes based on COG categories and CAZy families. (A)** Abundance of COGs of each COG functional category based on relative abundance of genes annotated per genome. **(B)** Abundance of CAZy families based on relative abundance of genes annotated per genome. The figures were drawn based on data shown in Table S1 **(A)** and Table 4 **(B)**.

For BZ6, the best 16S rRNA hit (98% identity) was to a partial sequence (accession FN667161.1) from an uncultured bacterium in a composting study in Finland (Partanen et al., 2010). We did not get any hits for the BLAST search using the DNA primase nucleotide sequence; using the translated sequence the best hit (66% identity) was to a Clostridiales bacterium (accession KKM09071.1). Phylogenetic analysis (Figure 1D) shows that BZ6 belongs to a divergent group of Clostridia: Clostridiales families XVII (*Thermaerobacter marianensis*) and XVIII (*Symbiobacterium thermophilum*) Incertae sedis. Currently these two families are classified as a sister group to other families in the order Clostridiales (Zhang and Lu, 2015). We hypothesize that the organism corresponding to BZ6 is a member of a new deep-branching family within the Clostridia class.

### Metabolic potential encoded by the reconstructed genomes

We analyzed the six reconstructed genomes using Cluster of Orthogous Groups, or COGs (Galperin et al., 2015; Figure 2A and Table S1). BZ4 presents the largest genome and therefore the highest number of CDSs (3,771) classified in COGs. CDSs classified as COG category G (metabolism and transport of carbohydrates) were more numerous in the BZ1 and BZ3 genomes, while CDSs classified as category E (amino acid metabolism and transport) were more numerous in the BZ4 and BZ6 genomes. Among the CDSs assigned to category G in BZ1 and BZ3, the most numerous had the following functional descriptions: xylanase, β-xylosidase, glycosidase, and arabinofuranosidase (Table 3). Genes encoding these predicted functions are likely playing a role in plant biomass biodegradation and some of them were also found in the BZ4 (xylanases and β-xylosidases) and BZ5 genomes (β-xylosidases). We remark that β-xylosidases are a key enzyme for the degradation of the main hemicellulose constituent, xylan, and relatively few xylosidases from thermophilic bacteria have been reported (Shao et al., 2011; Anand et al., 2013; Bhalla et al., 2014). Related to cellulose degradation, CDSs for two key enzymes were annotated: endo-1,4-beta-glucanase (cellulase M) in BZ2, BZ4, BZ5, and BZ6 and beta-glucosidase in BZ1, BZ3, BZ4, BZ5, and BZ6. CDSs encoding multicopper oxidases, which are involved in lignin breakdown, were also identified in BZ1, BZ2, BZ3, BZ4, and BZ6 (Table 3).

**Table 3:** **Number of CDSs assigned to COGs related to lignocellulose metabolism in reconstructed genomes**.

**COG number**	**CDS name**	**BZ1**	**BZ2**	**BZ3**	**BZ4**	**BZ5**	**BZ6**
COG0296	1,4-alpha-glucan branching enzyme	0	0	0	1	0	0
COG0366	Glycosidases	4	0	2	6	3	1
COG0383	Alpha-mannosidase	0	0	0	0	0	2
COG0438	Glycosyltransferase	13	8	11	7	4	10
COG0662	Mannose-6-phosphate isomerase	2	4	1	1	1	1
COG0726	Predicted xylanase/chitin deacetylase	8	0	7	7	4	2
COG0836	Mannose-1-phosphate guanylyltransferase	1	0	2	0	0	1
COG1172	Xylose/arabinose/galactoside ABC-type transport systems, permease components	4	1	3	1	1	10
COG1216	Predicted glycosyltransferases	2	2	3	1	0	0
COG1363	Cellulase M and related proteins	0	2	0	4	5	1
COG1440	Phosphotransferase system cellobiose-specific component IIB	0	0	0	0	6	0
COG1447	Phosphotransferase system cellobiose-specific component IIA	0	0	0	1	7	0
COG1455	Phosphotransferase system cellobiose-specific component IIC	0	0	0	1	5	0
COG1472	Beta-glucosidase-related glycosidases	3	1	1	1	1	0
COG1486	Alpha-galactosidases/6-phospho-beta-glucosidases, glycosyl hydrolases family 4	3	0	0	1	3	1
COG1501	Alpha-glucosidases, glycosyl hydrolases family 31	2	0	2	0	1	2
COG1874	Beta-galactosidase	4	1	1	0	0	0
COG2115	Xylose isomerase	1	0	1	1	1	1
COG2132	Putative multicopper oxidases	2	8	7	5	0	3
COG2152	Predicted glycosylase	1	2	0	1	1	1
COG2160	L-arabinose isomerase	2	0	1	0	0	0
COG2273	Beta-glucanase/Beta-glucan synthetase	1	0	0	0	0	0
COG2723	Beta-glucosidase/6-phospho-beta-glucosidase/beta-galactosidase	3	0	1	2	3	1
COG2730	Endoglucanase	0	0	0	1	0	0
COG2814	Arabinose efflux permease	21	19	18	18	8	7
COG3250	Beta-galactosidase/beta-glucuronidase	3	1	0	1	2	0
COG3345	Alpha-galactosidase	2	0	0	1	2	0
COG3405	Endoglucanase Y	1	0	1	0	0	0
COG3459	Cellobiose phosphorylase	3	0	0	0	5	0
COG3507	Beta-xylosidase	7	0	2	0	1	0
COG3534	Alpha-L-arabinofuranosidase	4	1	4	0	0	0
COG3661	Alpha-glucuronidase	1	0	1	1	0	0
COG3664	Beta-xylosidase	0	0	1	1	0	0
COG3693	Beta-1,4-xylanase	3	0	1	2	0	0
COG3858	Predicted glycosyl hydrolase	2	1	2	2	2	1
COG3867	Arabinogalactan endo-1,4-beta-galactosidase	1	0	0	0	0	0
COG3940	Predicted beta-xylosidase	1	0	0	0	0	0
COG4124	Beta-mannanase	0	0	1	0	0	0
COG4213	ABC-type xylose transport system, periplasmic component	3	0	3	1	0	0
COG4214	ABC-type xylose transport system, permease component	2	0	3	1	0	0
COG5520	O-Glycosyl hydrolase	1	1	0	0	0	0
COG5581	Predicted glycosyltransferase	2	1	2	1	1	1

### Lignocellulose breakdown potential viewed through cazy database

We screened the six reconstructed genomes for genes encoding lignocellulose-degrading enzymes using the CAZy database (Figure 2B). We found 691 different CAZyme genes, encompassing all six CAZy families, as follows: 33% glycoside hydrolases (GHs), 24% glycosyltransferases (GTs), 20% carbohydrate esterases (CEs), 1% polysaccharide lyases (PLs), 5% auxiliary activities (AAs), and 18% carbohydrate-binding modules (CBMs; Table 4).

**Table 4:** **Number of CDSs from reconstructed genomes mapped to enzymes in the CAZy database**.

**CAZy Class**	**Reconstructed genomes**						
	**CAZy Family**	**BZ1**	**BZ2**	**BZ3**	**BZ4**	**BZ5**	**BZ6**
SLH	–	34	13	35	29	0	4
GH	1	2	0	1	2	3	1
	2	3	1	0	1	2	0
	3	3	1	1	1	1	0
	4	3	2	0	1	3	1
	5	0	0	0	1	0	0
	8	1	0	1	0	0	0
	9	2	0	1	0	0	0
	10	3	0	1	2	0	0
	11	1	0	0	0	0	0
	13	4	0	2	8	3	1
	15	1	0	0	0	0	0
	16	1	0	0	0	0	0
	18	2	0	2	2	2	1
	23	2	2	1	2	1	3
	26	0	0	1	0	0	0
	30	1	1	0	0	0	0
	31	2	0	2	0	1	2
	32	1	4	0	1	1	0
	35	1	0	0	0	0	0
	36	2	0	0	1	2	0
	38	0	0	1	0	0	1
	39	0	0	1	1	0	0
	42	3	1	1	0	0	0
	43	9	0	3	0	1	0
	51	4	1	3	0	0	0
	52	0	0	0	1	0	1
	53	1	0	0	0	0	0
	57	0	0	0	0	0	1
	65	0	0	0	0	2	0
	67	1	0	0	1	0	0
	73	2	0	1	1	0	0
	74	0	0	0	1	0	0
	76	0	0	1	1	0	0
	78	0	0	0	1	0	1
	88	0	1	0	0	0	0
	94	2	0	0	0	2	0
	95	1	0	1	0	0	0
	105	4	0	1	0	0	0
	108	0	0	0	0	0	2
	109	13	2	11	3	5	7
	113	1	0	0	0	0	0
	115	0	0	2	0	0	0
	120	0	1	0	0	0	0
	127	2	0	1	0	0	0
	129	1	0	0	0	0	0
	130	1	2	0	1	1	1
GT	2	14	7	10	8	5	7
	4	12	7	10	5	4	7
	5	0	0	0	1	0	0
	8	1	0	1	2	0	0
	19	0	1	0	1	1	3
	26	1	3	2	1	1	2
	27	0	1	0	0	0	0
	28	2	3	2	4	4	1
	30	0	0	1	0	0	0
	32	1	0	0	0	0	0
	35	0	0	0	1	0	0
	39	0	0	1	1	0	0
	51	3	2	4	3	3	1
	81	0	1	0	2	0	2
	83	0	1	0	2	0	0
	84	1	0	0	0	1	0
	94	1	1	1	1	0	0
CE	1	14	6	4	6	5	3
	3	3	0	1	3	3	0
	4	8	7	8	7	4	2
	6	0	0	2	0	0	0
	7	2	1	0	4	1	0
	8	0	1	0	0	0	0
	9	3	0	2	1	1	1
	10	7	1	3	2	2	5
	11	0	0	0	0	0	1
	12	1	0	0	0	0	0
	14	2	1	2	2	1	2
	15	2	0	0	0	0	0
PL	9	2	0	1	0	0	0
	11	0	0	0	0	0	0
	12	0	1	0	0	0	1
	22	0	0	0	0	0	1
AA	1	0	1	0	0	0	0
	2	3	0	2	0	0	2
	4	0	2	2	5	1	2
	6	2	1	1	5	0	0
	7	0	0	1	0	0	2
CBM	6	4	0	1	0	0	0
	9	3	0	1	0	0	0
	16	0	0	0	0	1	1
	22	3	0	2	0	0	0
	25	0	0	0	0	0	1
	30	2	0	1	0	0	0
	32	0	0	5	0	0	0
	34	1	0	1	1	1	1
	35	1	0	0	0	0	0
	38	0	2	0	0	0	0
	40	0	1	0	0	0	0
	48	0	0	0	2	0	0
	50	13	9	15	17	14	12
	54	0	1	1	0	0	0
	61	2	0	0	0	0	0
	66	2	0	0	0	0	0

*CAZy families: GH, Glycoside Hydrolases; GT, GlycosylTransferases; CE, Carbohydrate Esterases; PL, Polysaccharide Lyases; AA, Auxiliary Activities; CBM, Carbohydrate-Binding Modules*.

The BZ1 genome shows the highest number of CDSs classified as CAZymes (196 CDSs, 6%) followed by BZ3 (128 CDSs, 4%; Figure 2B and Table 4). These numbers are within the range of CAZymes encoding-genes estimated for the genomes of thermophilic lignocellulose degraders such as *Thermobispora bispora DSM 43833* (181 CDSs, 5%), *Rhodothermus marinus* DSM 4252 (182 CDSs, 6.5%), and *Clostridium clariflavum* str. 4-2a (478, 12%; D'Haeseleer et al., 2013; Rooney et al., 2015; Hiras et al., 2016). BZ1 also stands out for being the richest in diversity and abundance of GHs compared to the other reconstructed genomes (80 CDSs, 35%). For instance, BZ1 has relatively more GHs than BZ4, which is the largest among the reconstructed genomes (BZ1: 0.02 per kbp; BZ4: 0.007 per kbp).

The other five species contribute collectively with 14 GH families not encoded in the BZ1 genome (GH5, GH26, GH38, GH39, GH52, GH57, GH65, GH74, GH76, GH78, GH88, GH108 and GH115, and GH120). Among GH families present in BZ1, GH109 is the most numerous (13 CDSs, 16%), followed by GH43 (9 CDSs, 11%). The GH109 family contains only the α-N-acetylgalactosaminidase enzyme. The GH43 family includes enzymes related to hemicellulose degradation such as β-xylosidase, α-L-arabinofuranosidase, arabinase, and xylanase. The BZ1 genome also presents three GH10 family enzymes, including a potential thermostable celloxylanase that can be involved with xylan degradation. CDSs assigned to cellulase families were found only in the BZ4 (GH5) and BZ1/BZ3 (GH9) genomes.

Overall, nine of the GH families (GH5, GH26, GH57, GH65, GH74, GH88, GH108, GH115, and GH120) are specific, occurring only in one of the five BZ genomes other than BZ1. For instance, the GH57 family (α-amylase) was identified only in the BZ6 genome (Table 4). Among these specific GH families, GH26 (in BZ3) is worth noting, because it includes exo-acting β-mannanase and β-1,3-xylanase, which are involved in hemicellulose degradation (Araki et al., 2000; Cartmell et al., 2008).

The BZ1 genome also encodes the greatest number of CBMs (25%), followed by BZ3 (22%), as computed with respect to all CDSs classified in this family, in the six BZ genomes. The most abundant family in all BZ genomes is CBM50 (80 CDSs, 65%; Table 4). CBM50 is a binding domain associated with chitin or peptidoglycan cleavage, present in several GH families such as GH18, GH23, and GH73. Accordingly, BZ4 and BZ5 encode one CDS classified as GH18 that contains a CBM50 domain. Three CBM families with members encoded in the reconstructed genomes are cellulose-binding (CBM9, CBM16, and CBM30) and one is xylan-binding (CBM22).

We found five AA families (AA1, AA2, AA4, AA6, and AA7) in the six reconstructed genomes. BZ4 possesses the highest number of CDSs assigned to this family (31%). AA4 and AA6 are the most abundant, representing 37 and 28%, respectively, of the total CDSs classified as an AA family. BZ3 and BZ6 encode a cellulose oxidase (AA7), and BZ1, BZ3, BZ4, and BZ6 have the highest number of lignin oxidases (AA1, AA2, AA4, and AA6). BZ2 and BZ5 have the smallest repertoire of genes encoding enzymes for cellulose and lignin oxidation.

### Consortium in action as revealed by thermophilic composting metagenomics and metatranscriptomics data

In a previous work, we studied the microbial community of the composting operation at the Sao Paulo Zoo Park (Antunes et al., 2016). Since the inoculum used to prepare the ZCTH02 consortium originated from a sample of that same composting operation, we had the possibility of verifying whether the genes we studied in this work were present during the actual composting process. First, we verified the presence of the BZ genomes in the metagenomic datasets obtained from the ZC4 composting cell. All BZ genomes except BZ2 had genome coverage values exceeding 70% in at least one sample; for BZ2 the maximum value observed was 38% (Table S2). We interpret these results as providing strong evidence for the presence of the organisms corresponding to the BZ genomes except BZ2 in composting cell ZC4. For BZ2 the evidence is weaker.

**Figure 3:**
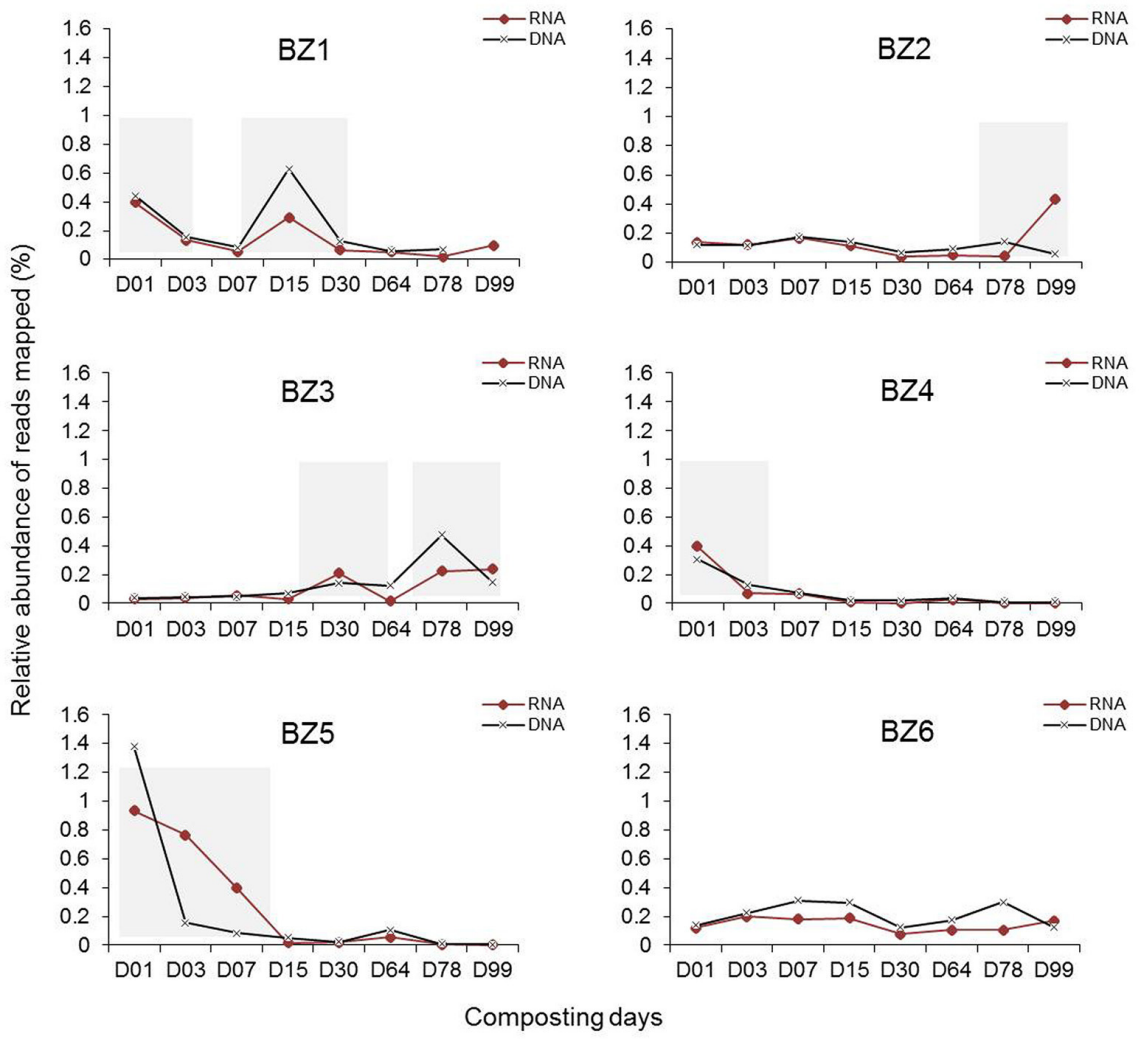
**Variation of metagenome and metatranscriptome reads mapped to the six reconstructed genomes over days of composting**. Relative abundance of reads (%) was calculated using total reads of each indicated genome per total reads in the metagenomic (DNA) or metatranscriptomic (RNA) sequences in samples collected from the Sao Paulo Zoo composting process and described previously (Antunes et al., 2016). Shaded bars indicate days of composting where the respective genomes were more abundant.

Next, we determined the relative abundance of the BZ genomes in the same metagenomic datasets as well as in the metatranscriptomic datasets (Table S3). The results are plotted in Figure 3. The variation between DNA and mRNA levels follows a similar pattern for each one of the reconstructed genomes, providing additional evidence for the presence of these organisms and evidence that they are active throughout the composting process. The number of metatranscriptomic reads mapping to BZ1 shows peaks in days 1 and 15 of composting. BZ2 has a peak in the last day (99), when the compost is considered mature. BZ3 appears more active in later stages, and BZ4 and BZ5 appear to be more active during initial stages (day 1 and until day 15, respectively). BZ6 seems to be present at moderate levels throughout the process.

**Figure 4:**
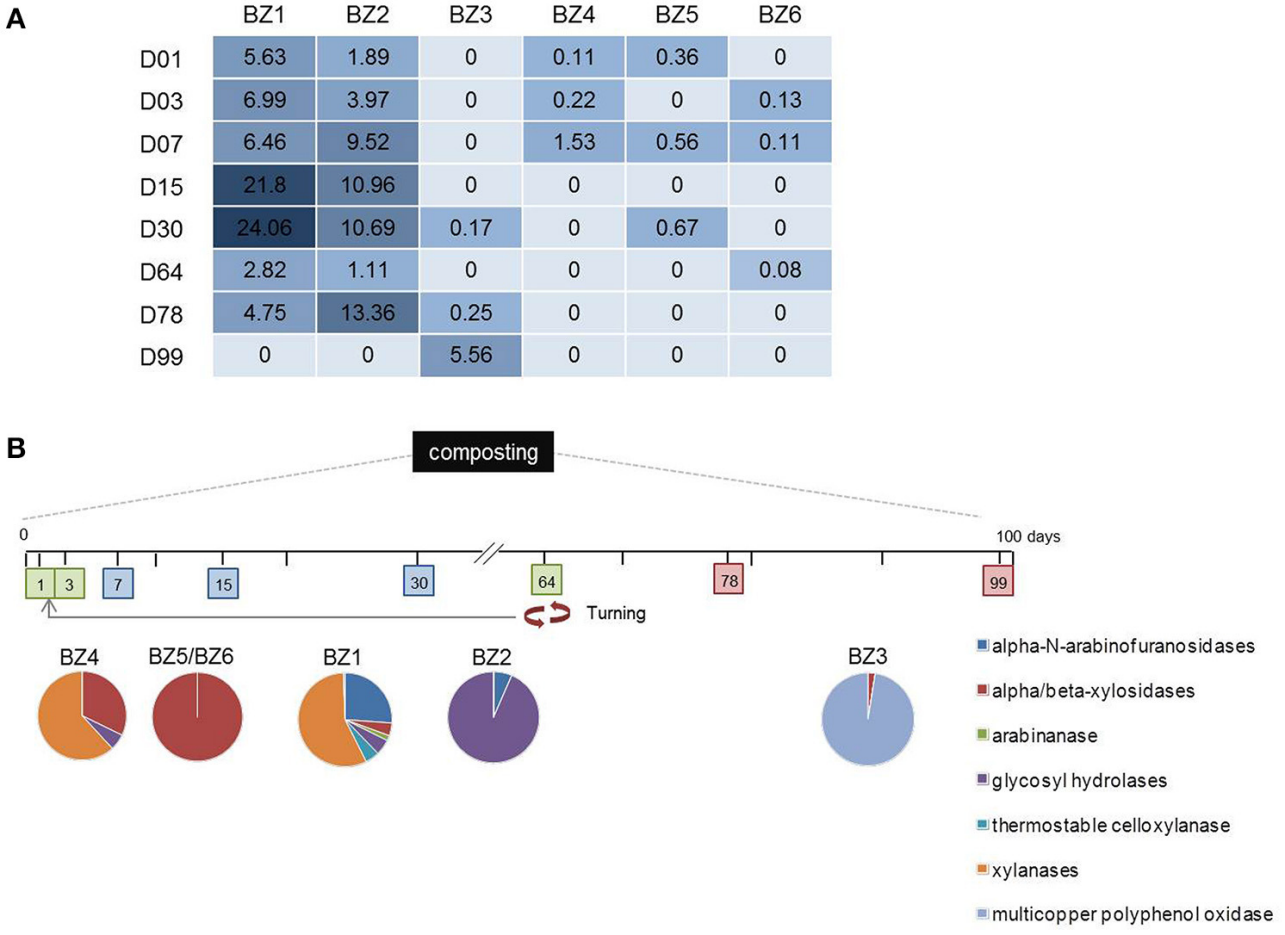
**Relative abundance of time-series composting metatranscriptome sequence reads mapped to lignocellulose degrading genes in the reconstructed genomes. (A)** The numbers in each day column refer to relative abundance of CDSs representing different enzymatic functions expressed in per thousand to which metatranscriptomic reads were mapped. **(B)** Colored pie charts show the amount of normalized reads mapped to lignocellulose-related enzyme genes from the six reconstructed genomes over days of thermophilic composting indicated in colored boxes. Red arrows indicate turning step (aeration of the composting pile).

We observed interesting correlations between the metabolic potential of the reconstructed genomes and the variation in their activity given by the time-series metatranscriptome mapping (Figure 4 and Table S4). On day 15, a point at which composting of easily degradable organic material is in full swing, a 1,4-beta xylanase is the fourth most abundant transcript mapped to the BZ1 genome. The mapping also shows BZ3 to be more active toward the middle and end phases of composting (days 30, 78, and 99). The fact that BZ3 encodes a multicopper oxidase, which is a lignin-degrading enzyme, and the fact that lignin becomes more accessible toward the latter stages of the composting might explain this correlation.

## Discussion

In this study, we used shotgun metagenomics to investigate a new thermophilic compost-derived consortium adapted for CMC digestion. This consortium was dominated by Firmicutes. The relative abundance of Firmicutes in diverse lignocellulose-degrading environments (Eichorst et al., 2014; Antunes et al., 2016; Güllert et al., 2016; Stolze et al., 2016; Zhu et al., 2016) underscores their importance and unique adaptation abilities. Previous studies on thermophilic aerobic lignocellulolytic consortia derived from green-waste composting have also shown the presence of Firmicutes, but in addition reveal the presence of other phyla, such as Bacteroidetes, Deinococcus-Thermus, and Actinobacteria, in varied proportions depending on the lignocellulosic substrate used for the enrichment (D'Haeseleer et al., 2013; Eichorst et al., 2013, 2014; Simmons et al., 2014a).

We were able to reconstruct six nearly-complete genomes from sequencing data obtained from this consortium, all of which were determined to be Firmicutes species. Four of these genomes are novel (new *Thermobacillus* species; the first *B. thermozeamaize* genome or else the first genome of a representative of a novel family in order Bacillales; a new genus of the Paenibacillaceae family and one new deep-branching family in the Clostridia class), and two are possibly new strains of *G. thermoglucosidasius* and *C. debilis*. In some cases, the reconstructed genomes belong to organism groups containing well-known biomass degraders or that play other roles in the biodegrading process. Members of the *Thermobacillus* genus are aerobic, and are known as plant biomass degraders, producing robust glycoside hydrolases and esterases that are of special interest for industrial applications (Wongwilaiwalin et al., 2010; Rakotoarivonina et al., 2012). *G. thermoglucosidasius* C56-YS93 is known as a biomass degrader and was isolated from the Obsidian hot spring in Yellowstone National Park (Brumm et al., 2015). A noncellulolytic facultative anaerobe *C. debilis* strain (GB1) isolated from an air-tolerant cellulolytic consortium has been studied for its ability to supply respiratory protection for cellulolytic bacteria, such as *Clostridium thermocellum*, when co-cultured (Wushke et al., 2015).

Regarding metabolic potential for lignocellulose degradation a diversity of cellulases (in families GH5 and GH9), endohemicellulases (GH8 and GH11), debranching enzymes (GH51 and GH67), and oligosaccharide-degrading enzymes (GH1, GH2, GH3, GH42, and GH43) were found in the reconstructed genomes. GH5 and GH9 include several enzymes related to cellulose and hemicellulose breakdown and have been described in biomass degrading metagenomes such as sugarcane bagasse (Mhuantong et al., 2015) and thermophilic composting (Antunes et al., 2016), as well as in some consortia enriched from composting (Allgaier et al., 2010; Wang et al., 2016). However, typical cellulases in the GH6 and GH48 families (endoglucanases and cellobiohydrolases) were not found to be encoded in the reconstructed genomes, a result consistent with a study of the bagasse microbiome (Mhuantong et al., 2015), which did not detect these families.

We identified BZ1 as a *Thermobacillus*. In the literature, the best studied *Thermobacillus* is *T. xylanilyticus* (Watanabe et al., 2007). *T. xylanilyticus* is a thermophilic and highly xylanolytic bacterium isolated from farm soil (Touzel et al., 2000; Paës and O'Donohue, 2006; Paës et al., 2008; Rakotoarivonina et al., 2011, 2012). In these reports, two enzymes were characterized: xylanase (GH11; Paës and O'Donohue, 2006) and α-L-arabinofuranosidase (GH51; Paës et al., 2008), both of which are present in the BZ1 genome. Another study (Rakotoarivonina et al., 2011) identified a hemicellulase enzyme (feruloyl-esterase) in *T. xylanilyticus*, adding evidence of the lignocellulose biomass degradation capabilities of this species. The most recent study reported that this species secretes a diverse arsenal of lignocellulolytic enzymes depending on the biomass composition used as carbon source (Rakotoarivonina et al., 2012). These studies were conducted with isolated strains. Therefore, this work is the first report of *Thermobacillus* in a thermophilic consortium.

**Figure 5:**
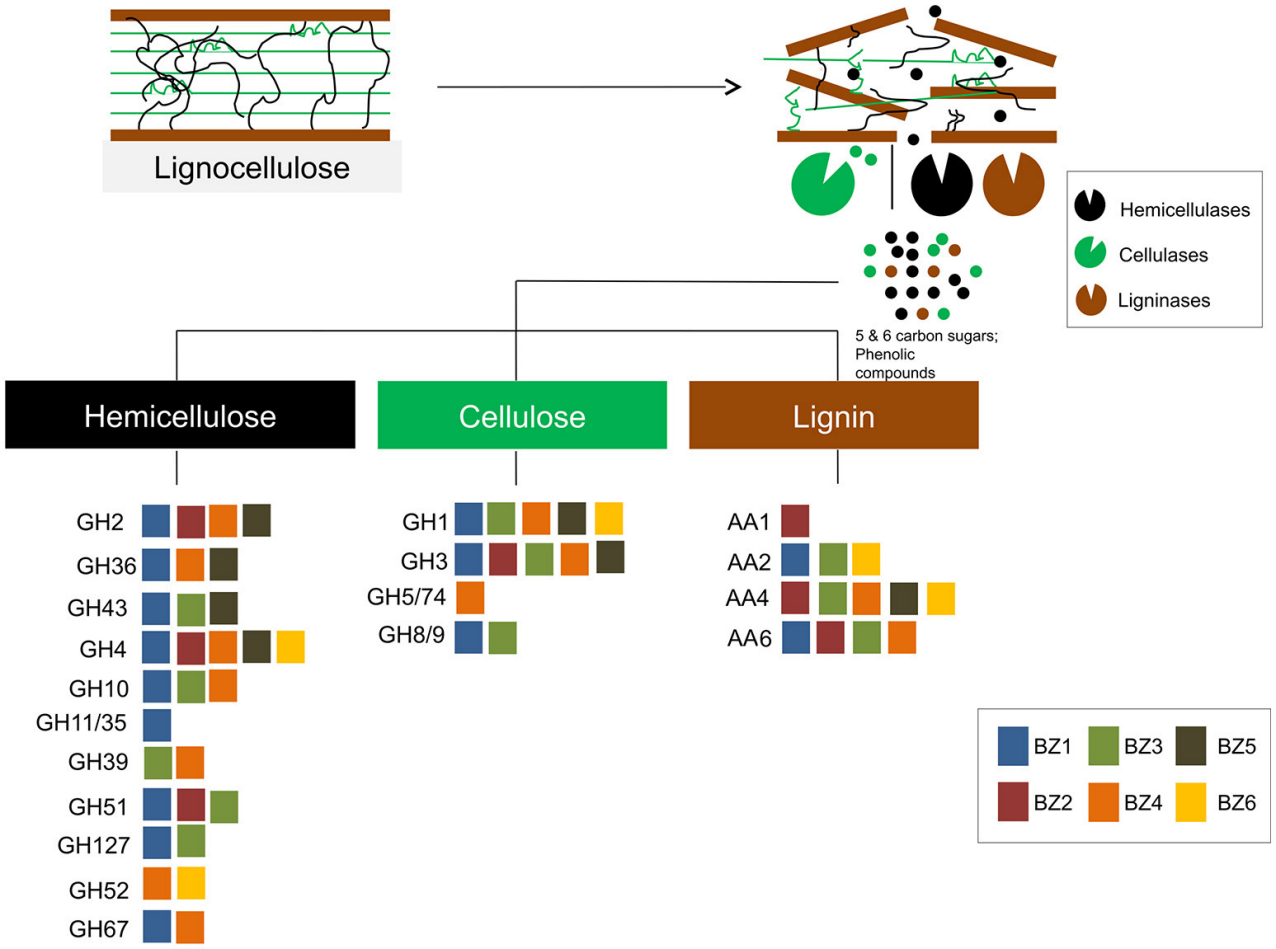
**An overview of lignocellulose degradation in the ZCTH02 consortium**. The diagram shows the main constituents of lignocellulose (hemicellulose, cellulose and lignin). Each colored square represents a GH family or an AA family that contains one or more CDSs that were annotated in a given BZ genome. The color of each square corresponds to the BZ genome where those CDSs were annotated, according to the key in the figure.

We found that the BZ1 genome encodes by far the largest number of biomass degrading-related genes as compared to the other genomes. For example, BZ1 encodes more than twice as many GH-classified CDSs as any single other genome (Figure 2B). This suggests that BZ1 has a primary role in lignocellulose breakdown in this consortium. On the other hand, there are 14 GH families for which the other genomes do have representatives while BZ1 has none. Based on this we speculate that BZ1 is a driver organism while the others are auxiliary in the lignocellulose breakdown process, in this consortium. Previous studies have reported that a variety of enzymes from different organisms can work together to improve lignocellulose degradation and access to energy sources (Takasuka et al., 2013; Hiras et al., 2016). Figure 5 summarizes these observations, showing an overview of lignocellulose degradation in the ZCTH02 consortium, depicting the distribution of GH and AA families among consortium component organisms.

We presented evidence that the organisms corresponding to the reconstructed genomes are present and active in a thermophilic composting process. However, none of the genomes reconstructed here were particularly abundant in the composting cell ZC4 (Antunes et al., 2016). This most likely has to do with the particular features of the consortium construction process, with its many selection steps. Aside from high temperature, most or all of the remaining selection features of the consortium construction process are very different from those of composting. This fact, coupled with the high compositional diversity of ZC4 (Antunes et al., 2016) probably explains why our consortium did not capture the most abundant species identified in ZC4.

Overall, our results illustrate the importance and plasticity of Firmicutes members in the bioconversion of lignocellulose in compost-derived consortium selected using CMC as carbon source. The large number and diversity of CAZyme genes investigated indicate that this consortium is composed by organisms that can complement each other with different enzymes relevant for lignocellulose degradation, being therefore a promising potential source for thermostable enzymes in industrial applications.

## Author contributions

Conceived the study and designed the experiments: AMdS, JS, RQ. Performed the experiments: LL, RQ. Processed the samples and performed DNA sequencing: LFM, LA, RQ. Analyzed the data: AMdS, ARdS, LFM, LL, LMSM, RQ, RP, JS. Wrote the manuscript: AMdS, LFM, LL, RP, JS.

## Funding

Funding for this research was provided by grant 2011/50870-6 from the São Paulo Research Foundation (FAPESP). LL, LA, and RP were supported by fellowships from FAPESP. LL received fellowship from FAPESP (2013/05325-5). ARdS and LMSM were supported by fellowships from the Coordination for the Improvement of Higher Education Personnel (CAPES). AMdS and JS received Research Fellowship Awards from National Council for Scientific and Technological Development (CNPq). The funders had no role in study design, data collection, analysis, decision to publish or preparation of the manuscript.

### Conflict of interest statement

The authors declare that the research was conducted in the absence of any commercial or financial relationships that could be construed as a potential conflict of interest.

## Footnotes

^1^https://github.com/aleimba/bac-genomics-scripts/tree/master/cdd2cog.

^2^https://www.biostarhandbook.com/tools/bbmap/bbmap-help.html.
